# Boosting toxic protein biosynthesis: transient in vivo inactivation of engineered bacterial alkaline phosphatase

**DOI:** 10.1186/s12934-020-01424-y

**Published:** 2020-08-18

**Authors:** Natalia Krawczun, Marta Bielawa, Kasjan Szemiako, Beata Łubkowska, Ireneusz Sobolewski, Agnieszka Zylicz-Stachula, Piotr M. Skowron

**Affiliations:** Department of Molecular Biotechnology, Faculty of Chemistry, University of Gdansk, Wita Stwosza 63, 80-308 Gdansk, Poland

## Abstract

**Background:**

The biotechnology production of enzymes is often troubled by the toxicity of the recombinant products of cloned and expressed genes, which interferes with the recombinant hosts’ metabolism. Various approaches have been taken to overcome these limitations, exemplified by tight control of recombinant genes or secretion of recombinant proteins. An industrial approach to protein production demands maximum possible yields of biosynthesized proteins, balanced with the recombinant host’s viability. Bacterial alkaline phosphatase (BAP) from *Escherichia coli* (*E. coli*) is a key enzyme used in protein/antibody detection and molecular cloning. As it removes terminal phosphate from DNA, RNA and deoxyribonucleoside triphosphates, it is used to lower self-ligated vectors’ background. The precursor enzyme contains a signal peptide at the N-terminus and is secreted to the *E. coli* periplasm. Then, the leader is clipped off and dimers are formed upon oxidation.

**Results:**

We present a novel approach to *phoA* gene cloning, engineering, expression, purification and reactivation of the transiently inactivated enzyme. The recombinant *bap* gene was modified by replacing a secretion leader coding section with a N-terminal His6-tag, cloned and expressed in *E. coli* in a *P*_*BAD*_ promoter expression vector. The gene expression was robust, resulting in accumulation of His6-BAP in the cytoplasm, exceeding 50% of total cellular proteins. The His6-BAP protein was harmless to the cells, as its natural toxicity was inhibited by the reducing environment within the *E. coli* cytoplasm, preventing formation of the active enzyme. A simple protocol based on precipitation and immobilized metal affinity chromatography (IMAC) purification yielded homogeneous protein, which was reactivated by dialysis into a redox buffer containing reduced and oxidized sulfhydryl group compounds, as well as the protein structure stabilizing cofactors Zn^2+^, Mg^2+^ and phosphate. The reconstituted His6-BAP exhibited high activity and was used to develop an efficient protocol for all types of DNA termini, including problematic ones (blunt, 3′-protruding).

**Conclusions:**

The developed method appears well suited for the industrial production of ultrapure BAP. Further, the method of transient inactivation of secreted toxic enzymes by conducting their biosynthesis in an inactive state in the cytoplasm, followed by in vitro reactivation, can be generally applied to other problematic proteins.

## Background

Alkaline phosphatases (APS) (EC 3.1.3.1) are enzymes commonly found in nature, from bacteria to mammals [1]. The major function of *E. coli* BAP is to supply a source of inorganic phosphate when the environment is deprived of this compound by increasing the rate of diffusion of this compound into the cells and preventing phosphate from leaving the cells [2]. Once a phosphate group is clipped from a variety of organic compounds, it needs to enter the cytoplasm. Gram-negative *E. coli* bacteria contain a double membrane, where the outer membrane is decorated with porin proteins, thus allowing for the diffusion of charged molecules. Since phosphate is a highly charged anion, *E. coli* utilises a dedicated permease for the transport of this ion through its internal membrane - a nonpolar region essentially impermeable to charged molecules. Such a dedicated system was described as the phosphate-specific transport system (Pst system) [3, 4]. APS are almost exclusively homodimeric metalloproteins. Their common architecture includes each catalytic site containing three metal ions: two Zn^2+^ and one Mg^2+^ [5, 6]. Furthermore, they require the adoption of a catalytically active conformation facilitated by disulfide bridges, among others. In the fully active, dimeric BAP, Zn^2+^ occupies active sites A and B, and Mg^2+^ occupies site C, thus the enzyme has the configuration (ZnAZnBMgC)2. Four cysteine residues create disulfide bridges linking the two subunits and are essential for mature BAP dimer activity [7]. Apparently, a combination of factors, including strong interactions between amino acid (aa) side chains, stabilisation of the 3D active conformation by divalent Zn^2+^ and Mg^2+^, as well as the presence of disulfide bridges result in an unexpected thermal stability of BAP, vastly exceeding the temperature growth range of *E. coli*. The enzyme is active up to 80 °C and even undergoes reversible renaturation at 90 °C, thus a heating step could be used in enzyme purification [5, 8]. This thermostability may play a role in the resistance of BAP to inactivation by harsh environmental conditions, present outside the cytosol. The enzymes exhibit wide substrate specificity, and catalyses: the hydrolysis of monoesters of phosphoric acid, including 5′ ends of DNA, RNA, nucleotides and a transphosphorylation reaction in the presence of high concentrations of phosphate acceptors. Moreover, it hydrolyses oxyphosphate monoesters [9, 10], as well as a variety of *O*- and *S*-phosphorothioates [11–13], phosphoramidates [10], thiophosphates and phosphates [14, 15]. A minor activity of *E. coli* BAP—oxidation of phosphite to phosphate was also detected. Purified BAP catalysed the oxidation of phosphite with specific activities approx. 1000-fold lower than phosphate ester hydrolysis. Interestingly, BAP catalyses the oxidation phosphite to phosphate and H_2_, thus it can be considered a phosphite-dependent hydrogenase that has emerged as a result of evolution [16]. Thus far, BAP was purified as a native or recombinant enzyme from its natural location in the periplasmic space, by weakening the outer membrane of cells, using for example osmotic shock [17] or a mutant *E. coli* strain [18]. BAP is widely used in molecular cloning for the removal of 5′ phosphates from linearized vectors, detecting PCR products, primer labelling and immunoassays. In this study, we describe a successful alternative strategy for the cloning and high production of BAP with transiently inhibited activity and thus ‘toxicity’ to the recombinant *E. coli* host. The strategy includes the biosynthesis of the leaderless His6-tagged BAP in the *E. coli* cytoplasm, followed by purification and oxidation/renaturation of the enzyme in vitro. We also believe that the developed method will be useful for the biotechnology scale production of other periplasm residing proteins/enzymes.

## Materials and methods

### Bacterial strains, plasmids, media and reagents

*Escherichia coli* DH5α {F– Φ80∆*lacZ*∆M15 ∆ (*lac*ZYA-*arg*F) U169 *rec*A1 *end*A1 *hsd*R17(r_K-_, m_K+_) *pho*A *sup*E44 λ-*thi*-*1, gyr*A96*, rel*A1} (Life Technologies, Gaithersburg, MD, USA) was used for electroporation and DNA propagation. *E. coli* HB101 {F^−^ *mcr*B *mrr hsd*S20(r_B_^−^ m_B_^−^) *rec*A13 *leu*B6 *ara*-14 *pro*A2 *lac*Y1 *gal*K2 *xyl*-5 *mtl*-1 *rps*L20(Sm^R^) *gln*V44 λ^−^} (Thermo Fisher Scientific (MA, USA)/GIBCO BRL) was used for wt *phoA* gene source. Bacteria were grown in 2xYT medium [19]. For protein biosynthesis *E. coli* TOP10 {F^−^ *mcr*A Δ(*mrr*-*hsd*RMS-*mcr*BC) φ80*lac*ZΔM15 Δ*lac*X74 *rec*A1 *ara*D139 Δ(*ara*-*leu*)7697 *gal*U *gal*K *rps*L(Str^R^) *end*A1 *nup*G λ^−^} was used (Invitrogen, CA, USA). The bacteria were cultivated in Terrific Broth (TB) medium [19], supplemented with ampicillin (100 µg/ml) and 0.2% maltose. Difco media components were obtained from Becton–Dickinson (Franklin Lakes, NJ). The proofreading Pwo Polymerase and DNA purification kits were from BLIRT (Gdansk, Poland). BsaI, NcoI and HindIII restriction endonucleases (REases) were from New England Biolabs (Ipswich, MA, USA). Protein standards, 100 bp DNA, 1 kb DNA markers, the cloning vector pBADmycHisA (Ap^R^, colE1 *ori*, *P*_*BAD*_ promoter) and Pierce™ BCA Protein Assay Kit were from Thermo Fisher Scientific. The DNA sequencing and PCR primer synthesis were conducted at Genomed (Warsaw, Poland). Ni Sepharose 6 Fast Flow chromatographic resin (GE Healthcare, cat. no GE17-5318-06), glutathione oxidized form (cat. no G4626), SIGMAFAST™ Protease Inhibitor Tablets (cat. no S8820), SIGMAFAST™ p-Nitrophenyl phosphate (pNPP) tablets (cat. no N2770), alkaline phosphatase from *E. coli* (cat. no P4252), reagents for Glycine with Zinc Enzymatic Assay and all the other chemicals were from Sigma-Aldrich (St. Louis, MO, USA).

### Cloning of modified *his6*-*phoA* gene

Modified *his6*-*phoA* gene, encoding leaderless BAP protein was PCR amplified from the *E. coli* genome. The PCR reactions were performed in 50 μl samples in a thermocycler (Applied Biosystems, CA, USA) and contained: 1 × Pwo PCR Buffer, 0.2 mM of each dNTP, 2 μM of each primer, 100 ng *E. coli* HB101 genomic DNA, 1 mM MgCl_2_ and 1 unit of Pwo DNA polymerase. Mutagenic primers (Table 1) were used for PCR.

**Table 1:** DNA sequence of PCR primers used for *his6*-*phoA* gene cloning and engineering

Primer name	DNA sequence	Target
FAlkPHisBsaI	5′-CCCCGGTCTCTC*ATG*CCAATGTCTCACCACCA TCACCACCAT**AGAACACCAGAAATGCCTGT**-3′	wt *phoA* gene GenBank M29663.1 protein id AAA24363.1
RAlkPHindIII	5′-CACGCCGGGCAAGCTT*TTA***TTTCAGCC**-3′	wt phoA gene GenBank M29663.1 protein id AAA24363.1

The introduced BsaI and HindIII restriction sites are underlined. DNA fragments introducing restriction sites, an aminopeptidase protection segment and His6-tag are written in capital letters. DNA fragments complementary to the leaderless wt *phoA* gene are in bold capital letters. Start and Stop codons are in italics. The PCR cycling profile was as follows: 94 °C for 3 min, 80 °C for 20 s (addition of Pwo DNA polymerase), 94 °C for 30 s, 51 °C for 30 s, and 72 °C for 90 s (for 30 cycles); 72 °C for 3 min. PCR products were purified and digested with BsaI and HindIII. A Type IIS REase - BsaI - in this case generated 4-nt cohesive ends compatible with NcoI cohesive ends. An arabinose-regulated expression vector pBADmycHisA [20] was cut with NcoI and HindIII. Both the insert and the vector were separated using 1.2% agarose gel electrophoresis, purified and subjected to ligation using T4 DNA ligase at 16 °C overnight. The resulting DNA was used to transform *E.coli* DH5α competent cells. After electroporation, the bacteria were plated onto 2xYT medium supplemented with ampicillin (100 µg/ml) and incubated at 28 °C. The selection of positive bacterial clones was conducted by PCR screening of bacterial colonies using original mutagenic primers. After a preliminary analysis, plasmid DNAs isolated from the selected bacterial clones were subjected to DNA sequencing.

### Expression of the recombinant *his6*-*phoA* gene under *P*_*BAD*_ promoter in *E. coli*

The resulting positive bacterial clones were subjected to recombinant gene expression experiments. *E. coli* BL21(DE3) were electroporated with pBAD_BAP1 and mini-scale expression was performed by cultivation in 50 ml TB media supplemented with 100 μg/ml of ampicillin, at 28 °C with vigorous aeration. *P*_*BAD*_ promoter induction was performed by the addition of 0.2% arabinose, when A_600_ reached 0.8. The culture growth was continued for 19 hours (h) at 37 °C. Bacterial pellets from both the control, non-induced and induced cultures were subjected to SDS/PAGE electrophoresis. The gels were analysed for the appearance of the expected band size of ~ 40 kDa and for colour reaction using the p-nitrophenol assay. The bacterial clones, efficiently expressing the *his6*-*phoA* gene, were selected for large-scale bacterial culture. Scaling up for biotechnology production included: (*i*) cultivation of *E. coli* TOP10[pBAD_BAP1] in 0.5 L media containing 100 μg/ml ampicillin in a 5 L Erlenmeyer flasks at 37 °C with vigorous aeration (200 rpm) until A_600_ reached 0.5 and arabinose was added to 0.02% for the induction of the *P*_*BAD*_ promoter. The flasks were further shaken for 18 h; (*ii*) cultivation of *E. coli* TOP10[pBAD_BAP1] in 5 L TB media containing ampicillin at 100 μg/ml in a New Brunswick Scientific BioFlo115 fermenter at 28 °C with vigorous aeration until A_600_ reached 1.0, then arabinose was added to 0.02% and further cultivation was conducted for 5 h at 37 °C.

### Purification and reactivation of the recombinant His6-BAP enzyme

The recombinant His6-BAP purification procedure employed a simple protocol, which included: (*i*) removal of nucleic acids and acidic proteins with polyethyleneimine (PEI); (*ii*) ammonium sulphate fractionation and (*iii*) metal affinity purification using Ni Sepharose 6 Fast Flow with immobilised Ni^2+^ ions.

#### *E. coli* cells lysate preparation

Recombinant *E. coli* TOP10[pBAD_BAP1] cells (2.5 g) were spun down and resuspended in 25 ml cold lysis buffer [30 mM Tris–HCl pH 8.0 at 20 °C, 30 mM NaCl, 5% glycerol, 3 mM 2-mercaptoethanol (βME), protease inhibitors]. Lysozyme was added to 0.5 mg/ml and the suspension was incubated for 30 min at 4 °C. Solid NaCl was added to a final 250 mM concentration. Then, the initially lysed suspension was sonicated for 15 × 1 min pulses with 1 min breaks at 0 °C (ice bath), until no increase of A_280_ was observed in samples taken after every sonication pulse. Finally the cell debris was spun down at 19,650 × g at 4 °C for 30 min.

#### PEI and ammonium sulphate fractionation

To the supernatant, 10% (v/v) PEI solution (pH 7.5) was slowly added until a final 1% (v/v) solution was obtained and the suspension was stirred for 30 min. The precipitated complexes of PEI-nucleic acids and PEI-acidic proteins were spun down at 19,650 x g at 4 °C for 30 min. To the supernatant, solid ammonium sulphate was added at 0.5 g/ml and stirred overnight at 4 °C. Precipitated proteins were spun down at 19,650 x g at 4 °C for 30 min, the supernatant discarded and the protein pellet dissolved in 30 ml of buffer N [50 mM K/PO_4_ pH 8.0, 20 mM imidazole, 5% glycerol, 3 mM βME, 0.02% Triton X-100, protease inhibitors]. The remaining undissolved proteins were centrifuged and discarded.

#### Immobilized metal affinity purification (IMAC) using Ni Sepharose 6 Fast Flow

Previous purification steps removed the bulk of the cellular contaminants from the crude His6-BAP-containing cell lysate. This allowed us to take full advantage of the high specificity of the immobilised Ni^2+^ interaction with His6-tagged recombinant BAP. The precipitated proteins after ammonium sulphate fractionation were dissolved in 30 ml of N1 buffer [20 mM K/PO_4_ pH 8.0, 20 mM imidazole, 5% glycerol, 3 mM βME, 0.02% Triton X-100; protease inhibitors] and undissolved proteins were removed by centrifugation. The preparation was loaded onto a 4 ml column, packed with Ni Sepharose 6 Fast Flow, equilibrated in buffer N20 [20 mM K/PO_4_ pH 8.0, 20 mM imidazole, 0.5 M NaCl, 5% glycerol, 3 mM βME, 0.02% Triton X-100; protease inhibitors], washed with 20 ml of buffer N20, followed by washing with 20 ml of buffer N40 [20 mM K/PO_4_ pH 8.0, 40 mM imidazole, 0.5 M NaCl, 5% glycerol, 3 mM βME, 0.02% Triton X-100; protease inhibitors] and eluted with  12 ml of buffer N500 [20 mM K/PO_4_ pH 8.0, 500 mM imidazole, 0.5 M NaCl, 5% glycerol, 3 mM βME, 0.02% Triton X-100; protease inhibitors]. The BAP purification protocol was scaled up for test purification from 50 g of the induced cells and the same results were obtained.

#### His_6_-BAP enzymatic activity reactivation

The purified preparation was dialysed overnight at 4 °C against two changes of 1 L reactivation-oxidation buffer [20 mM K/PO_4_ pH 7.0, 100 mM KCl, 0.2 mM MgCl_2_, 0.2 mM ZnCl_2_, 10% glycerol, 0.05% Tween 20, 0.05% Nonidet 40, 5 mM oxidized glutathione, 0.1 mM βME]. For the oxidation reaction, glutathione (oxidized form) is used from a fresh 0.5 M stock in water (stored at − 80 °C). To the dialysed His6-BAP preparation, oxidized glutathione was added to increase its final concentration to 20 mM and incubated overnight at 37 °C. The next day, the enzyme was dialyzed overnight at 4 °C against 1 L of storage buffer S [100 mM KCl, 0.1 mM MgCl_2_, 0.1 mM ZnCl_2_, 0.05% Tween 20, 0.05% Nonidet 40, 20 mM Tris–HCl pH 7.0 at 22 °C, 5 mM K/PO_4_, pH 7.0, 5 mM oxidized glutathione, 0.1 mM βME, 50% glycerol] and stored at –20 °C.

### Spectrophotometric assay for His_6_-BAP enzymatic activity

The colorimetric assay of purified His_6_-BAP was conducted as based on the rate of release of p-nitrophenol from p-nitrophenyl phosphate by following the absorbance at 410 nm [5, 21]. One unit (U) was defined as the activity releasing 1 μM p-nitrophenol per minute at 25 °C in 3 ml of reaction buffer containing: 1 mM p-nitrophenyl phosphate, 1 M Tris–HCl, pH 8.0 at 25 °C. The U calculation (specific activity) was as follows: U/mg protein = ^A/min × 1000/1.62 × 10,000 × mg enzyme/ml reaction [22].

### Enzymatic assay of alkaline phosphatase

A comparative enzymatic ‘Glycine with Zinc Assay’ of purified His_6_-BAP and commercially available alkaline phosphatase from *E. coli* (Sigma-Aldrich) was conducted as based on the rate of release of p-nitrophenol from p-nitrophenyl phosphate by following the absorbance at 405 nm. One unit will hydrolyze 1.0 μmol of p-nitrophenyl phosphate per minute at pH 10.4 at 37 °C. Final assay concentrations: In a 1.50 ml reaction mix, the final concentrations are 87 mM glycine, 0.90 mM magnesium chloride, 0.87 mM zinc chloride, 6.0 mM p-nitrophenyl phosphate and 0.01–0.02 units of alkaline phosphatase. The U calculation (specific activity) was as follows: U/ml enzyme $$= \frac{{(\Delta {\text{A}}405{\text{nm}}/\hbox{min} Test - \Delta {\text{A}}405{\text{nm}}/\hbox{min} Blank)\left( {volume of assay} \right)\left( {dilution factor} \right)}}{{\left( {{\text{millimolar extinction coefficient of p}} - {\text{nitrophenolat }}405 {\text{nm}}} \right)\left( {\text{volume of enzyme used}} \right)}}$$ [23].

### His6-BAP functional assays

To assess the His6-BAP capability to efficiently dephosphorylate DNA ends, assays were conducted in parallel with the highest quality commercial preparation that we could locate (Sigma, P4252). The Sigma BAP was sold as an ammonium sulphate precipitate suspension. 280 μl suspension was carefully mixed, 100 μl (35.7 U) was removed, centrifuged at 14000 x g at 4 °C for 15 min and the supernatant was discarded. The Pellet was dissolved in 178.5 μl of buffer A [200 mM KCl, 0.2 mM MgCl_2_, 0.2 mM ZnCl_2_, 40 mM Tris–HCl pH 6.8 at 22 °C, 50% glycerol], obtaining an enzyme solution of concentration 0.2 U/μl. Ten-fold serial dilutions of both Sigma BAP and His6-BAP were made in buffer A. Each reaction mixture in a 60 μl volume of reaction buffer R [50 mM Tris–HCl pH 8.0 at 55 °C, 1 mM MgCl_2_, 0.1 mM ZnCl_2_] contained 1 pmol (app. 1.6 μg) of either SmaI-linearized or KpnI-linearized or EcoRI linearized pUC19 vector and a serial dilution of the enzymes. REase-digested pUC19 DNA was purified using a DNA Clean-Up kit prior to the dephosphorylation reaction. Following incubation at 55 °C for 45 min, the reactions were terminated by purification using a DNA Clean-Up kit and self-ligated in a 30 μl ligation buffer supplemented with 5% PEG4000, using 10 Weiss U of T4 DNA ligase. The reactions were terminated by heating at 70 °C for 5 min and the addition of SDS-containing loading dye/buffer and subjected to 1.0% agarose gel electrophoresis in TAE buffer. Gels were stained with ethidium bromide and photographed.

### Gel electrophoresis and protein concentration determination

Agarose gels (1.0%) for DNA analysis were prepared in TAE buffer [19]. The gels were visualized after staining with ethidium bromide using a 312 nm UV transilluminator or after staining with SYBR Green I using a 312 nm UV transilluminator and photographed with a SYBR Green gel stain photographic filter. SDS-PAGE electrophoresis of the proteins was in 10% polyacrylamide gels [19]. The protein concentrations were measured with a Pierce™ BCA Protein Assay Kit according to the manufacturer’s instructions.

## Results

### Rationale, design and cloning of a *his6*-*phoA* gene from *E. coli*

**Fig. 1:**
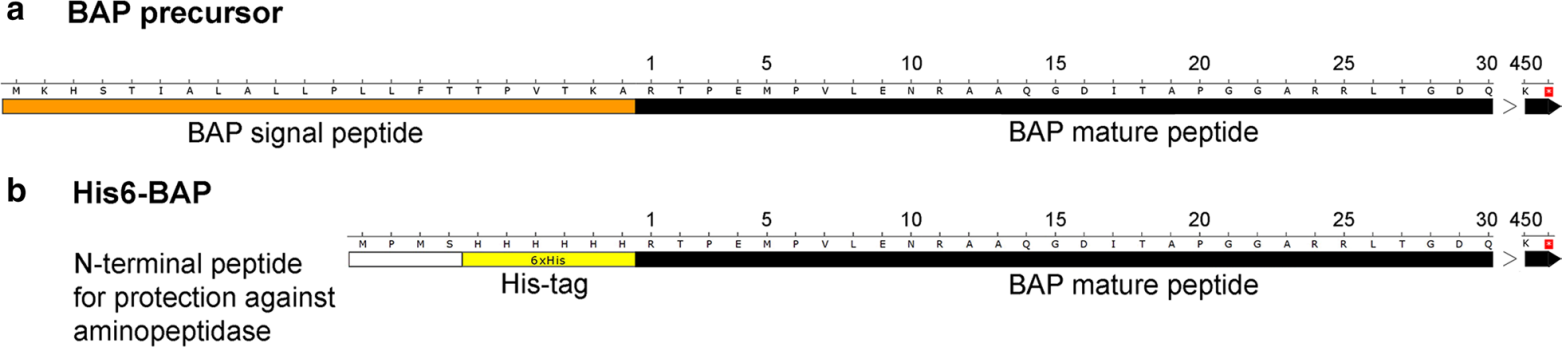
Differences between the aa sequences of the wt BAP precursor and the engineered, leaderless His6-BAP polypeptide. The N-terminal aa sequence of the alkaline phosphatase precursor (GenBank M29663.1; protein id AAA24363.1). **b** The N-terminal aa sequence of the 48.4 kDa recombinant His6-BAP protein

The rationale behind the general concept to obtain high overproduction of a toxic, recombinant protein was to biosynthesize it in a transiently inactive state: in the cytoplasm, which provides a reducing environment. This was to be followed by refolding in vitro under mild conditions in an oxidizing environment in the presence of a reaction product or substrate, redox buffer and cofactors, if applicable. In the example shown in this work, the modified His6-BAP was devoid of a secretion leader. Thus the produced truncated pre-protein was prevented from entering the periplasmatic space, which is required for disulfide bridge formation. Upon isolation from the cytoplasm of recombinant *E. coli*, the truncated, modified His6-BAP was to be restored to the enzymatically active state by dialysis into a buffer with pH and ionic strength simulating in vivo conditions. For this purpose, a mixture of oxidised and reduced sulfhydryl group-containing organic compounds, the protein structure stabilizing divalent ions cofactors Zn^2+^ and Mg^2+^ and dephosphorylation reaction product – phosphate was used. The native *phoA* 3′ gene fragment of 1353 bp, coding for a leaderless C-terminal ORF and a Stop codon was PCR amplified with a mutagenic forward primer (62-nt) (Table 1), containing an overhang (42 nt) and mismatches, which introduced the BsaI restriction site. This arrangement removed the *phoA* leader-coding a 63 bp (21 aa) 5′ region, replacing it with an in-frame 18 bp segment coding for: 6 histidine residues for IMAC and a 4 aa segment MPMS, protecting the His6-tag from aminopeptidases in vivo and adding more immunogenic properties to the His6-tag for the possibility of using custom made antibodies (Fig. 1). The reverse mutagenic primer (27-nt) introduced a HindIII site. Following amplification, the reaction product was cut with BsaI and HindIII, purified and cloned into NcoI-HindIII cleaved arabinose-regulated pBADmycHisA expression vector in a perfect fusion with the vector’s ATG codon. Since BsaI is a Type IIS REase, its 4-nt generated cohesive ends can be of any sequence. Thus they made CATG-5′ to be compatible with the NcoI-generated cohesive ends. The resulting constructs were sequenced from both ORF ends and the pBAD_BAP1 clone was selected for expression experiments (Additional files 1, 2, 3, 4). The His6-BAP ORF was of 1383 bp, coding for a 460 aa fusion protein of molecular weight of 48.5 kDa and theoretical pI of 5.89 compared to native, mature BAP (450 aa, 47.2 kDa, pI of 5.54) and wt BAP (471 aa, 49.5 kDa, pI of 5.81). The theoretical pIs were calculated using the ProtParam tool (https://web.expasy.org/protparam/).

### Overproduction, purification and His6-BAP activity reconstitution in vitro

**Fig. 2:**
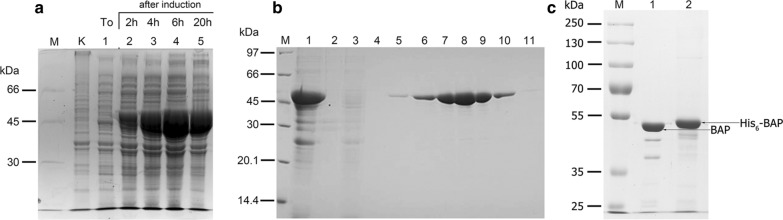
Biosynthesis and isolation of the recombinant His6-BAP protein from *E. coli*. **a** Overproduction of the recombinant His6-BAP in induced *E. coli* TOP10[pBAD-BAP1]. Lane M, LMW-SDS Marker Kit (GE Healthcare, cat. no 17044601); lane K, crude extract from *E. coli* TOP10; lane 1, *E. coli* TOP10[pBAD_BAP1] before induction; lanes 2–5, *E. coli* TOP10[pBAD_BAP1] after induction: 2 h, 4 h, 6 h and 20 h. **b** His6-BAP isolation and purification. Lane M, protein marker (Thermo Fisher Scientific, cat. no 26610); lane 1, extract from the induced *E. coli* TOP10[pBAD_BAP1] cells after PEI and ammonium sulphate treatment; lane 2, the metal affinity column flow-through; lanes 3 and 4, column wash; lanes 5–11, elution of homogeneous His6-BAP. Panel c. Comparative electrophoresis of His6-BAP and Sigma BAP. lane 1, Sigma BAP; lane 2, His6-BAP; Lane M, protein marker (Thermo Fisher Scientific, cat. no. 26619)

In rich media and under intense aeration, the arabinose-induced recombinant *E. coli* TOP10 [pBAD_BAP1] produced massive amounts of His6-BAP, being the predominant band on SDS-PAGE gels. Cultivation of up to 20 h resulted in the highest His6-BAP accumulation after 6 h (Fig. 2a). Figure 2a shows the time course of induction, where whole *E. coli* TOP10 [pBAD_BAP1] cells were lysed in denaturing buffer and loaded onto SDS-PAGE. Since the 4^th^ h after induction, His6-BAP comprises the majority of cellular proteins. Thus, taken together the high biosynthesis level and His6-tag, a simple 4-step purification protocol was devised, comprising of: (*i*) sonication of entire cells, instead of extracting BAP from the periplasm; (*ii*) PEI precipitation of nucleic acids and acidic proteins; (*iii*) ammonium sulphate fractionation and (*iv*) IMAC. Purifications steps (*ii*) and (*iii*) removed the bulk of the cellular contaminants from the crude His6-BAP-containing cell extract. This allowed us to take full advantage of high specificity of the immobilised Ni^2+^ interaction with the His6-tagged recombinant His6-BAP. The interaction was strong, as 40 mM imidazole-containing elution buffer eluted only trace amounts of the His6-BAP, while the bulk of the His6-BAP eluted as a sharp peak with 500 mM imidazole (Fig. 2b). Overall, the purification stages (*ii*)-(*iv*), each based on a different principle, were sufficient to obtain a homogeneous protein (Fig. 2b). The step (*ii*) of PEI precipitation of nucleic acids was also selected as beneficial from the stand point of application in genomic library preparations, as no *E. coli* DNA should be carried over with His6-BAP to be used for dephosphorylation. To increase yields and for protective purposes, glycerol and non-ionic detergents were added to block hydrophobic patches on the His6-BAP protein surface and prevent the protein from denaturation, aggregation and adhesion. Optionally, considering the reported thermostability of BAP [5, 8], a heat treatment step can be added. While heat treatment would not increase the apparent purity of the preparation, when examined on SDS-PAGE gel, for some special purposes it can be included into the presented purification protocol after ammonium sulphate fractionation and prior to IMAC (not shown). As judged by SDS-PAGE, fractions containing the highest His6-BAP content (Fig. 2b, lanes 6–10) were pooled and dialysed against the oxidizing buffer to promote the folding of the enzyme into an active state. Next, the enzyme was dialysed against storage buffer, containing 50% glycerol and all the components of reactivation-oxidation buffer to maintain a stable redox environment. The buffered glycerol preparation was stored for over 4 years at − 20 °C without an apparent loss of activity (not shown). The final preparation was assayed in a standard colorimetric assay using p-nitrophenyl phosphate [5, 21, 22] in parallel with several available commercial preparations and their relative activities were compared. The His6-BAP exhibited a specific activity of approx. 80% of the commercially available preparations of the highest purity (not shown). Figure 2c shows the comparison of two BAP preparations: Sigma BAP (Fig. 2c, lane 1) and His6-BAP (Fig. 2c, lane 2), run in parallel as overloaded bands. The His6-BAP enzyme is stable, with minimal signs of degradation, when stored for over 4 years in a dedicated storage buffer with 50% glycerol (Fig. 2c, lane 2). Due to the presence of the His6-tag, the His6-BAP has a higher molecular weight than Sigma BAP. The purity of both enzymes is very similar, with two more minor bands observed in the Sigma BAP preparation.

### Evaluation of His6-BAP performance in DNA dephosphorylation for molecular cloning

**Fig. 3:**
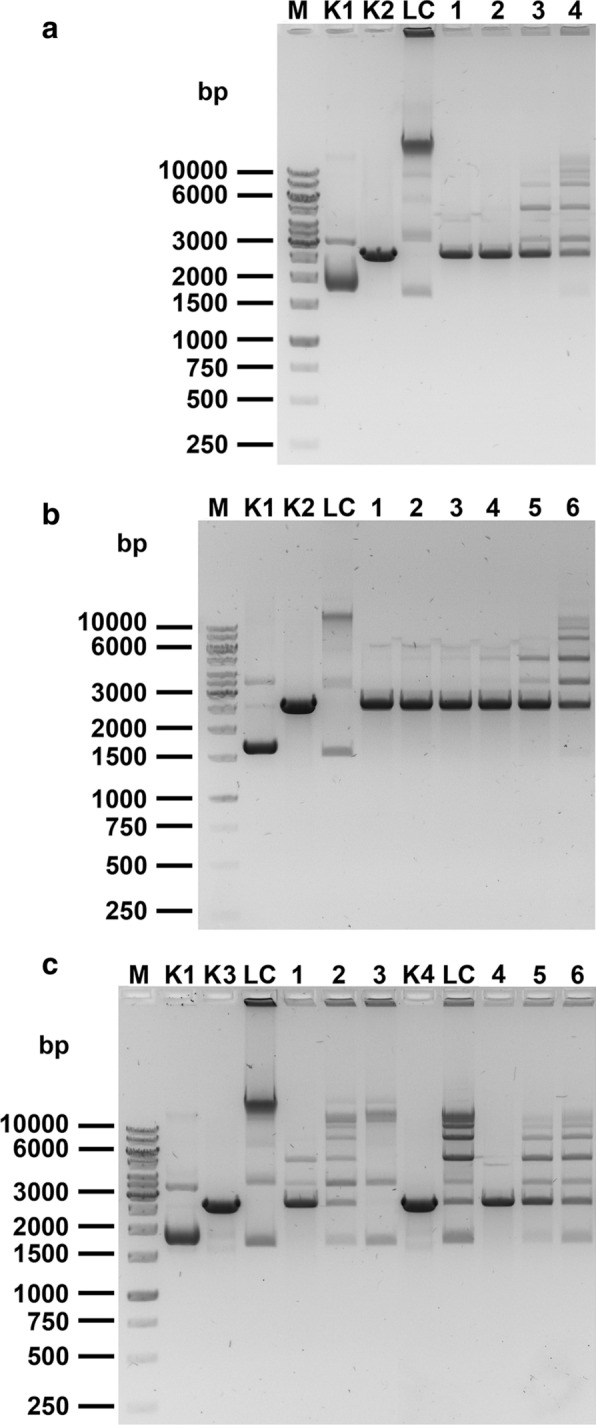
Functional pUC19 vector dephosphorylation assays. **a**. EcoRI-linearized pUC19 DNA, subjected to dephosphorylation and self-ligation. Lane M, GeneRuler 1 kb DNA Ladder; lane K1, undigested pUC19 DNA; lane K2, EcoRI-linearized pUC19 DNA; lane LC (ligation control), non-dephosphorylated, selfligated EcoRI-linearized pUC19 DNA; lane 1, EcoRI-linearized pUC19 DNA dephosphorylated with 0.01 colorimetric U of His6-BAP and selfligated; lane 2, with 0.005 U; lane 3, with 0.0025 U; lane 4, with 00125 U. **b** EcoRI-linearized pUC19 DNA, subjected to dephosphorylation and self-ligation—fine-tuned His6-BAP concentrations. Lane M, GeneRuler 1 kb DNA Ladder; lane K1, undigested pUC19 DNA; lane K2, EcoRI-linearized pUC19 DNA; lane LC, non-dephosphorylated, selfligated EcoRI-linearized pUC19 DNA; lane 1, EcoRI-linearized pUC19 DNA dephosphorylated with 0.006 colorimetric U of His6-BAP and selfligated; lane 2, with 0.005 U; lane 3, with 0.004 U; lane 4, with 0.003 U; lane 5, with 0.002 U; lane 6, with 0.001 U. **c** KpnI- or SmaI-linearized pUC19 DNA, subjected to dephosphorylation and self-ligation. Lane M, GeneRuler 1 kb DNA Ladder; lanes K1, undigested pUC19 DNA; lane K3, KpnI-linearized pUC19 DNA; lane LC, non-dephosphorylated, selfligated KpnI-linearized pUC19 DNA; lane 1, KpnI-linearized pUC19 DNA subjected to dephosphorylation with 0.05 colorimetric U of His6-BAP and selfligation; lane 2, as in lane 1, except that 0.01 U His6-BAP were used; lane 3, 0.005 U; lane K4, SmaI-linearized pUC19 DNA; lane LC, non-dephosphorylated, selfligated SmaI-linearized pUC19 DNA; lane 4, SmaI-linearized pUC19 DNA dephosphorylated with 0.05 colorimetric U of His6-BAP and selfligated; lane 5, with 0.01 U; lane 6, with 0.005 U

One of the major purposes of this work was to provide large amounts of ultra-pure alkaline phosphatase for molecular cloning approaches, where thermolabile phosphatases do not perform well, namely, in preparation of blunt- and protruding 3′ termini of vector’s DNA. For this purpose, we tested the His6-BAP in a vector dephosphorylation assay. Figure 3 shows the general cloning vector pUC19 cleavage/dephosphorylation/self-ligation assay. Clearly, His6-BAP efficiently dephosphorylates the vector’s DNA. In the final range tested (from 5x10^−3^ to 5x10^−2^ colorimetric units) both Sigma BAP and His6-BAP efficiently dephosphorylated KpnI-generated 3′ sticky ends and SmaI-generated blunt ends. For cloning, it is more convenient to use a DNA dephosphorylation activity unit, which is different than the definition of the activity unit used in the classical p-nitrophenyl phosphate assay. One should note that the phosphatase unit definition may vary, depending on manufacturer, but in general it is based on dephosphorylation of a set amount of DNA (such as 1 μg) or pmols of 5′ends (such as 1 pmol) in a set reaction time, temperature and buffer composition. As 1 pmol of DNA ends is a little over about 1 µg of a 3 kb plasmid, these definitions are not far away from each other. Typically they use 10–30 min reaction time and 37 °C. For the purpose of this work we have adopted and modified the unit definition used by New England Biolabs [24]. Thus, after testing a number of variables (not shown) and considering the time effectiveness of cloning procedures, we selected a convenient practical ‘cloning unit’ for His6-BAP. The following conditions/definitions were used: dephosphorylation of 1 pmol of SmaI-linearized 3 kb plasmid DNA for 45 min at 55 °C, in the His6-BAP dephosphorylation buffer. Thus, one colorimetric unit corresponds to 20 DNA dephosphorylation units (‘cloning units’) for blunt/protruding 3' DNA termini as determined in Fig. 3c, lanes 1,4. In the case of dephosphorylation of 5' protruding DNA termini, app. 10 times higher activity was observed (Fig. 3b, lanes 3-4). Even though the temperature of 55 °C was used, BAP thermostability allows for a further increase of the reaction temperature, if needed. When less enzyme is used, one can observe a mixture of ligated and non-ligated plasmid forms, indicating than only partial dephosphorylation was obtained. For the 3′-protruding, KpnI-linearized pUC19 DNA, very similar results were obtained, with slightly lower dephosphorylation efficiency, as faint ligated bands can be observed (Fig. 3c, lane 1). Thus, for a maximum reduction of the cloning background, we recommend to use an  excess of His6-BAP, namely a minimum of 5-10 ‘cloning units’ per 1 μg of the linearized plasmid of approx. 3 kb. The same results were obtained both for Sigma BAP and His6-BAP.

## Discussion

Here, we propose a novel approach to the high level biosynthesis of toxic proteins by expressing them in an inactive state. This is followed by the use of an in vitro gentle reactivation method, not employing harsh protein unfolding compounds, such as guanidinium chloride, but rather based on a tertiary structure recovery stimulation by enzyme cofactors, reaction substrates, products and disulfide bridge formation in a buffer resembling in vivo conditions. Further, this apparently allowed correct arrangement of disulfide bridges formation. All of these factors help the distant, catalytically active, polypeptide segments to come together in a native arrangement. This is an alternative and/or complementary approach to our previous publications concerning the expression of high GC content toxic genes. In that work we designed, synthesized and cloned the entire recombinant genes, encoding thermophilic restriction endonucleases-methyltransferases (REases-MTases), ‘toxic’ to a recombinant *E. coli* host. For this purpose we used a modified ‘one amino acid-one codon’ optimization method combined with weighting toward low GC content codons. This approach allowed for a significant expression increase of a thermophile gene in the recombinant host [25]. Together with the post-optimization sequence scanning for mRNA secondary structures, codon clusters and the local codon environment, the final synthetic gene became ‘*E. coli* friendly’, allowing for a one-order of magnitude increase in *taqIIRM* gene expression. In another approach, we used a biased ‘codon randomization’ method, which besides ORF optimization, apparently sped up the translation kinetics, which proved to be critically important to allow recombinant TthHB101RM REase-MTase to fold into an active state [26]. All the above approaches, including His6-BAP, can be combined to efficiently biosynthesize secreted ‘toxic’ proteins from a thermophile or other bacteria/archaea with GC-rich genomes. As the expression of the wt *phoA* gene is induced in *E. coli* (when grown under phosphate-limiting conditions) and results in moderate production of BAP, it can be considered as already naturally ‘optimized’ with regard to codon usage context, mRNA sequence and structure among other factors. Thus there was no need for applying gene optimisation procedures to obtain a high expression level, when natural regulation of *phoA* gene was replaced by the vector’s precisely regulated *P*_*BAD*_ promoter [20], as we obtained in our hands as massive natural codon-containing gene expression as for best optimised. This system is approaching the levels of expression as known from highest robustness T7-lac promoter-containing expression vectors [27]. The native BAP gene, containing a secretion leader, was cloned previously into bacteriophage lambda and further subcloned into the pBR322 vector derivative [28, 29] and into a derivative of pBluescript-SK(-) vector [30]. Those constructs were not for overproduction and directed BAP into the periplasm. However, an interesting observations were made by others that concern *E. coli* BAP production [31] on overproduction of the cloned native BAP precursor, where formation of cytoplasmic inclusion bodies was observed. Accumulation of BAP precursor at a high rate indicates that its synthesis can be uncoupled from secretion [31]. It may also suggest, that the presence of leader secretion peptide in the BAP precursor impairs its folding, thus leading to the inclusion bodies formation and providing additional natural route of transient toxicity suppression. In contrast, we cloned the mature, leaderless BAP with added His6-tag. The recombinant His6-BAP was biosynthesized in the *E. coli* cytoplasm in an soluble, but inactive form, thus non-toxic. This allowed for massive biosynthesis, comprising over 50% of cellular proteins of the recombinant host. We believe that this strategy will be useful for biotechnology industry production. The isolation/purification is fairly straightforward, with only one chromatographic step on Ni^2+^-chelating metal affinity resin, proceeded by two rapid precipitation steps involving PEI and ammonium sulphate, yielding a homogeneous protein. Further, we developed a simplified and mild BAP reactivation protocol, based on oxidation of cysteines in the presence of correct fold-stimulating agents (in this case Mg^2+^, Zn^2+^, phosphate, detergents, physiological pH and salt concentration). While native (= secreted and purified from periplasm) BAP was subjected to denaturation and successful refolding into an active state previously, these approaches were based on harsh conditions, such as the use of concentrated solutions of guanidinium hydrochloride [32–34]. Further, in those previous works, concerning denaturation/renaturation of phosphatases, there were used natively matured enzymes, thus already containing disulfide bridges in correct arrangements [32–34]. The overproduction, purification and reactivation procedure of His6-BAP is easily scalable. We repeated the described procedure in various variants, using from 0.5 g up to 50 g of the induced bacterial cells. Comparative assays of the His6-BAP (conducted in parallel with the highest activity available commercially BAP preparation) showed that His6-BAP has  essentially the same specific activity. Previously it was found that BAP is a thermostable enzyme in a mesophilic, cytoplasmatic environment [18]. This does not necessarily mean that there is an evolutionary relationship with ‘truly’ thermophilic enzymes, rather points to convergent protein structure evolution of mesophilic enzyme to withstand the harsher than cytoplasmatic conditions of an external environment. This feature can be exploited in adding an extra purification step to the His6-BAP protocol presented here, if desired for special application purposes. The thermal stability of BAP imposes linearized vector DNA purification upon dephosphorylation. Using other thermolabile phosphatases, such as calf intestinal phosphatase [35] or oceanic shrimp phosphatase [36], is convenient in DNA manipulations, as they can be heat-inactivated. However, high BAP thermostability may be of practical use in DNA manipulation methodologies in some applications, such as dephosphorylation of GC-rich blunt ends of DNA molecules, as DNA end ‘breathing’ at elevated temperatures makes 5′-phosphates more accessible to the enzyme.

## Conclusions

1. *E. coli phoA* gene was engineered by removal of the secretion leader and its replacement by a His6-tag and cloned under the *P*_*BAD*_ promoter control, which resulted in the cytoplasmatic biosynthesis of the recombinant His6-BAP.
2. A very high level of inactive His6-BAP overproduction was achieved as a result of combination of the strong *P*_*BAD*_ promoter and transient suppression of His6-BAP toxicity by preventing essential disulfide bridge formation in the reducing cytoplasmatic environment.
3. A simple protocol of purification to homogeneity of His6-BAP, suitable for biotechnology production, was developed.
4. An in vitro His6-BAP activity reactivation protocol was developed, based on folding and gentle oxidation in redox buffer, supplemented with cofactors (Zn^2+^, Mg^2+^), reaction product (phosphate) and non-ionic detergents.
5. An efficient dephosphorylation protocol was developed for all types of DNA termini: blunt, 3′ protruding and 5′ protruding.

## Supplementary information

**Additional file 1.** Nucleotide and aa sequences of the His6-BAP ORF. The genetic map was prepared using SnapGene software version 4.1 (http://www.snapgene.com).

**Additional file 2.** Map of pBAD_BAP1.

**Additional file 3.** Map, nucleotide and aa sequences of the recombinant pBAD_BAP1 construct. The additional file was prepared using SnapGene software version 4.1 (http://www.snapgene.com).

**Additional file 4.** Nucleotide sequence of the recombinant pBAD_BAP1 construct.

## Data Availability

All the data needed to repeat experiments are present in the manuscript and supplementary files. His6-BAP clones are available upon request.
