# Supplementary material for: Boosting toxic protein biosynthesis: transient in vivo inactivation of engineered bacterial alkaline phosphatase
Source: Microb Cell Fact. 2020 Aug 18;19:166. doi: 10.1186/s12934-020-01424-y (PMC7437050; doi:10.1186/s12934-020-01424-y)
Supplement: Supplementary file 1 — Additional file 1. Nucleotide and aa sequences of the His6-BAP ORF. The genetic map was prepared using SnapGene software version 4.1 (http://www.snapgene.com). [file 12934_2020_1424_MOESM1_ESM.pdf]

Sequence: His6_BAP.dna (Linear / 1383 bp)
Enzymes: Unique 6+ Cutters (37 of 666 total)
Features: 1 total

Unique Cutters **Bold**

Start (0) · **BsgI** · **Bpu10I** · **BsiHKAI** · **Bsp1286I** · **BsrBI**

5′ atgCCAATGTCTCACCACCATCACCACCATAGAACACCAGAAATGCCTGTTCTGGAAAACCGGGCTGCTCAGGGCGATATTACTGCACCCGGCGGTGCTCGCCGTTTAACGGGTGATCAGACTGCCGCTCTGCGTGATTCTCTTAGCGATAAACC 155
3′ tacGGTTACAGAGTGGTGGTAGTGGTGGTATCTTGTGGTCTTTACGGACAAGACCTTTTGGCCCGACGAGTCCCGCTATAATGACGTGGGCCGCCACGAGCGGCAAATTGCCCACTAGTCTGACGGCGAGACGCACTAAGAGAATCGCTATTTGG

1 5 10 15 20 25 30 35 40 45 50
M P M S H H H H H H R T P E M P V L E N R A A Q G D I T A P G G A R R L T G D Q T A A L R D S L S D K P

6xHis · BAP mature peptide

His6_BAP

BfuAI · **BspMI** · **SspI** · **BtgZI** · **BsaAI** · **DraI**

TGCAAAAAATATTATTTTGCTGATTGGCGATGGGATGGGGGATTCGGAAATTACTGCCGCACGTAATTATGCCGAAGGTGCGGGCGGCTTTTTTAAAGGTATCGATGCCTTACCGCTTACCGGGCAATACACTCACTATGCGCTGAATAAAAAAA 310
ACGTTTTTTATAATAAAACGACTAACCGCTACCCTACCCCCTAAGCCTTTAATGACGGCGTGCATTAATACGGCTTCCACGCCCGCCGAAAAAATTTCCATAGCTACGGAATGGCGAATGGCCCGTTATGTGAGTGATACGCGACTTATTTTTTT

55 60 65 70 75 80 85 90 95 100
A K N I I L L I G D G M G D S E I T A A R N Y A E G A G G F F K G I D A L P L T G Q Y T H Y A L N K K

BAP mature peptide

His6_BAP

NmeAIII · **PflFI** · **Tth111I** · **HincII** · **BssHII** · **BseYI** · **PspFI** · **BsaHI** · **Bsu36I**

CCGGCAAACCGGACTACGTCACCGACTCGGCTGCATCAGCAACCGCCTGGTCAACCGGTGTCAAAACCTATAACGGCGCGCTGGGCGTCGATATTCACGAAAAAGATCACCCAACGATTCTGGAAATGGCAAAAGCCTCAGGTCTGGCGACCGGT 465
GGCCGTTTGGCCTGATGCAGTGGCTGAGCCGACGTAGTCGTTGGCGGACCAGTTGGCCACAGTTTTGGATATTGCCGCGCGACCCGCAGCTATAAGTGCTTTTTCTAGTGGGTTGCTAAGACCTTTACCGTTTTCGGAGTCCAGACCGCTGGCCA

105 110 115 120 125 130 135 140 145 150 155
T G K P D Y V T D S A A S A T A W S T G V K T Y N G A L G V D I H E K D H P T I L E M A K A S G L A T G

BAP mature peptide

His6_BAP

AclI · **RsrII** · **EciI**

AACGTTTCTACCGCAGAGTTGCAGGATGCCACGCCCGCTGCGCTGGTGGCGCATGTGACCTCGCGCAAATGCTACGGTCCGAGCGCGACCAGTGAAAAATGTCCGGGTAACGCTCTGGAAAAAGGCGGAAAAGGATCGATTACCGAACAGCTGCT 620
TTGCAAAGATGGCGTCTCAACGTCCTACGGTGCGGGCGACGCGACCACCGCGTACACTGGAGCGCGTTTACGATGCCAGGCTCGCGCTGGTCACTTTTTACAGGCCCATTGCGAGACCTTTTTCCGCCTTTTCCTAGCTAATGGCTTGTCGACGA

160 165 170 175 180 185 190 195 200 205
N V S T A E L Q D A T P A A L V A H V T S R K C Y G P S A T S E K C P G N A L E K G G K G S I T E Q L L

BAP mature peptide

His6_BAP

BssSαI · **SgrAI**

TAACGCTCGTGCCGACGTTACGCTTGGCGGTGGCGCAAAAACCTTTGCTGAAACGGCAACCGCCGGTGAATGGCAGGGAAAAACGCTGCGTGAACAGGCACAGGCGCGTGGTTATCAGTTGGTGAACGATGCCGCCTCGCTAAATGCGGTGACGG 775
ATTGCGAGCACGGCTGCAATGCGAACCGCCACCGCGTTTTTGGAAACGACTTTGCCGTTGGCGGCCACTTACCGTCCCTTTTTGCGACGCACTTGTCCGTGTCCGCGCACCAATAGTCAACCACTTGCTACGGCGGAGCGATTTACGCCACTGCC

210 215 220 225 230 235 240 245 250 255
N A R A D V T L G G G A K T F A E T A T A G E W Q G K T L R E Q A Q A R G Y Q L V N D A A S L N A V T

BAP mature peptide

His6_BAP

BstXI · **BtgI** · **StyI** · **NcoI**

AAGCGAATCAGCAAAAACCCCTGCTTGGCCTGTTTGCTGACGGCAATATGCCAGTGCGCTGGCTAGGACCGAAAGCAACGTACCATGGCAATATCGATAAGCCCGCAGTCACCTGTACGCCTAATCCGCAACGTAATGACAGTGTACCAACCCTG 930
TTCGCTTAGTCGTTTTTGGGGACGAACCGGACAAACGACTGCCGTTATACGGTCACGCGACCGATCCTGGCTTTCGTTGCATGGTACCGTTATAGCTATTCGGGCGTCAGTGGACATGCGGATTAGGCGTTGCATTACTGTCACATGGTTGGGAC

260 265 270 275 280 285 290 295 300 305 310
E A N Q Q K P L L G L F A D G N M P V R W L G P K A T Y H G N I D K P A V T C T P N P Q R N D S V P T L

BAP mature peptide

His6_BAP

Esp3I
BsmBI

```
GCGCAGATGACCGACAAAGCCATTGAATTGTTGAGTAAAAATGAGAAAGGCTTTTTCCTGCAAGTTGAAGGTGCATCAATCGATAAACAGGATCACGCTGCGAATCCTTGTGGGCAAATTGGCGAGACGGTCGATCTCGACGAAGCCGTACAACG   1085
CGCGTCTACTGGCTGTTTCGGTAACTTAACAACTCATTTTTACTCTTTCCGAAAAAGGACGTTCAACTTCCACGTAGTTAGCTATTTGTCCTAGTGCGACGCTTAGGAACACCCGTTTAACCGCTCTGCCAGCTAGAGCTGCTTCGGCATGTTGC
```

315 320 325 330 335 340 345 350 355 360

A Q M T D K A I E L L S K N E K G F F L Q V E G A S I D K Q D H A A N P C G Q I G E T V D L D E A V Q R

BAP mature peptide

His6_BAP

ApoI
EcoRI
EcoNI
BtsaI

```
GGCGCTGGAATTCGCTAAAAAGGATGGCAACACGCTGGTCATAGTCACCGCTGATCACGCCCACGCCAGCCAGATTGTTGCGCCGGACACCAAAGCGCCGGGCCTCACCCAGGCGCTAAATACCAAAGATGGCGCAGTGATGGTGATGAGTTACG   1240
CCGCGACCTTAAGCGATTTTTCCTACCGTTGTGCGACCAGTATCAGTGGCGACTAGTGCGGGTGCGGTCGGTCTAACAACGCGGCCTGTGGTTTCGCGGCCCGGAGTGGGTCCGCGATTTATGGTTTCTACCGCGTCACTACCACTACTCAATGC
```

365 370 375 380 385 390 395 400 405 410

A L E F A K K D G N T L V I V T A D H A H A S Q I V A P D T K A P G L T Q A L N T K D G A V M V M S Y

BAP mature peptide

His6_BAP

MmeI
PasI
End (1383)

```
GGAACTCCGAAGAGGATTCACAAGAACATACCGGTAGTCAGCTGCGTATTGCGGCGTATGGCCCACATGCCGCCAATGTCGTTGGACTGACCGACCAGACCGATCTCTTCTACACCATGAAAGCCGCCCTGGGGCTGAAATAA   3′
                                                                                                                                                       1383
CCTTGAGGCTTCTCCTAAGTGTTCTTGTATGGCCATCAGTCGACGCATAACGCCGCATACCGGGTGTACGGCGGTTACAGCAACCTGACTGGCTGGTCTGGCTAGAGAAGATGTGGTACTTTCGGCGGGACCCCGACTTTATT   5′
```

415 420 425 430 435 440 445 450 455 460

G N S E E D S Q E H T G S Q L R I A A Y G P H A A N V V G L T D Q T D L F Y T M K A A L G L K *

BAP mature peptide

His6_BAP
