# Supplementary figures and images for: Boosting toxic protein biosynthesis: transient in vivo inactivation of engineered bacterial alkaline phosphatase
Source: Microb Cell Fact. 2020 Aug 18;19:166. doi: 10.1186/s12934-020-01424-y (PMC7437050; doi:10.1186/s12934-020-01424-y)

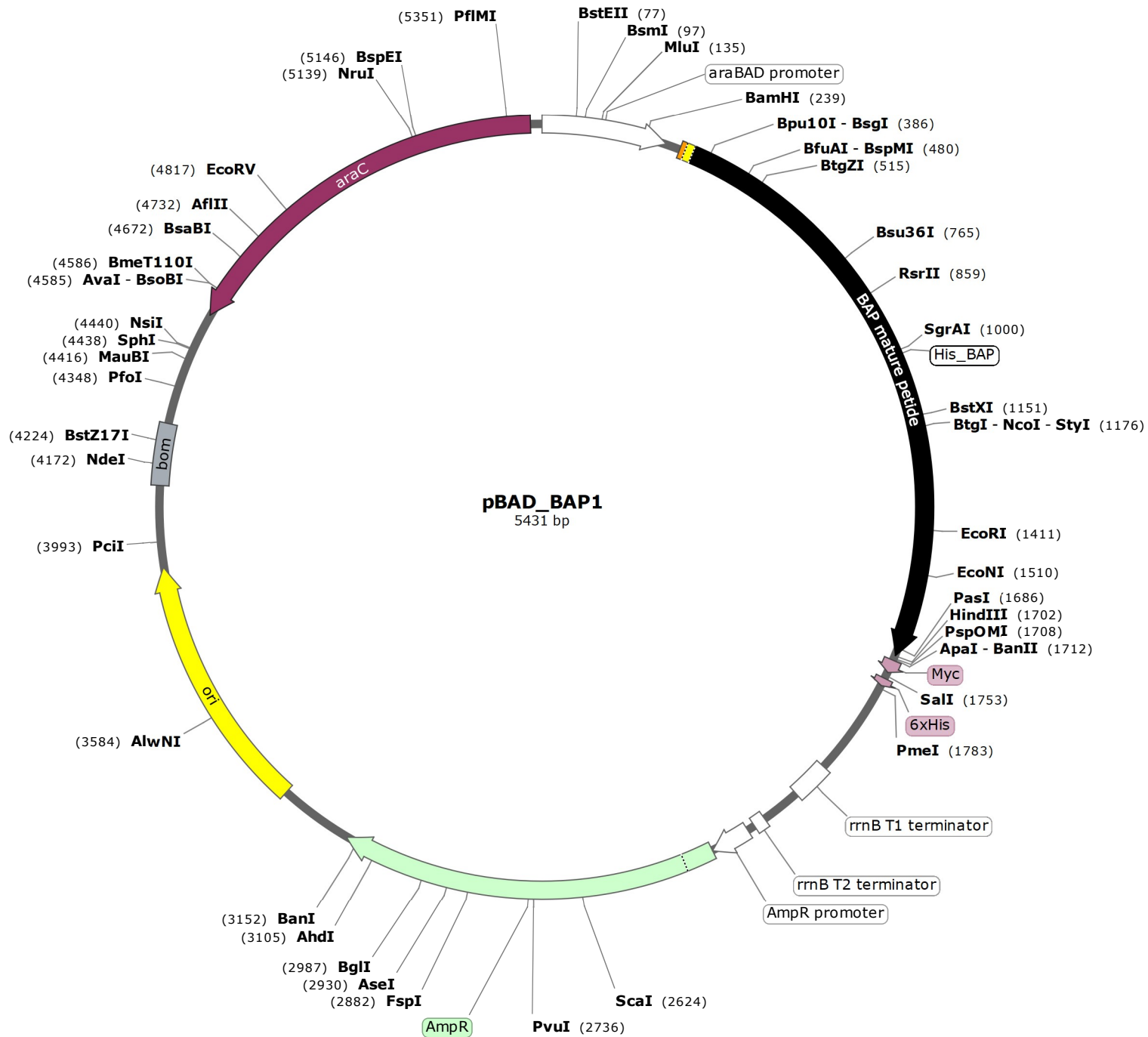

Supplement: Supplementary file 2 — Additional file 2. Map of pBAD_BAP1. [file 12934_2020_1424_MOESM2_ESM.pdf]
